# How do interdisciplinary practitioners conceptualize infection control in nursing homes during the COVID-19 pandemic?

**DOI:** 10.1080/17482631.2026.2658927

**Published:** 2026-04-13

**Authors:** Sung Ok Chang, Min Sun Park

**Affiliations:** a College of Nursing, Korea University, Seoul, Republic of Korea; b Department of Nursing, College of Health & Medical Sciences, Cheongju University, Cheongju, Republic of Korea

**Keywords:** Infection control, nursing homes, phenomenography, interdisciplinary communication, education, qualitative research

## Abstract

**Aim:**

During the recent global pandemic, nursing home (NH) residents were among the most vulnerable, yet empirical insights into how practitioners conceptualized and implemented infection control in NHs were limited. This study aimed to explore the ways in which interdisciplinary NH practitioners understood and conceptualized infection control in response to emerging infectious diseases and to identify a structural framework derived from their perceptions.

**Design:**

A qualitative phenomenographic research design was employed.

**Methods:**

In-depth interviews were conducted with 21 interdisciplinary practitioners from three NHs. Data were analyzed using the seven-step phenomenographic approach.

**Results:**

Two overarching categories were identified: “assessments based on vulnerability” and “interventions focused on prevention,” each consisting of sub-categories that reflected practitioners’ ways of understanding infection control. A hierarchical outcome space was developed to illustrate the dynamic and cyclical nature of infection control in NHs.

**Conclusion:**

The findings highlight the need for a proactive, interdisciplinary approach tailored to the unique environment of NHs. This framework may guide future educational initiatives and provide a practical reference for training new practitioners lacking pandemic experience.

## Introduction

Since its emergence, coronavirus disease 2019 (COVID-19) has continued to affect populations globally, resulting in widespread infection and considerable mortality (Centres for Disease Control & Prevention, 2023; World Health Organisation, 2024). The pandemic has placed a significant burden on long-term care systems, particularly in nursing homes (NHs), which have emerged as highly vulnerable settings due to their communal living arrangements and medically complex resident populations (Konetzka et al., 2021). NHs serve as essential long-term residential care facilities, primarily supporting older adults with cognitive and physical impairments (Lee et al., 2024a). Within Korea’s long-term care insurance system, nursing homes are officially defined as non-medical welfare facilities offering long-term services for older adults, particularly those living with dementia (National Health Insurance Service, 2023).

NH residents often experience severe physical and cognitive decline, leading to a high level of dependency on caregivers for activities of daily living (Lee et al., 2024a). Many display behavioural and psychological symptoms of dementia (BPSD), making it difficult to adhere to infection prevention protocols (Cheon et al., 2022; Kim et al., 2024).

These limitations, coupled with reduced mobility and impaired comprehension of safety protocols, significantly increase the risk of infection transmission through contact or respiratory routes (Centres for Disease Control & Prevention, 2021). In these settings, effective infection control involves not only preventing transmission but also preserving residents’ remaining functional abilities and quality of life. Therefore, infection management in NHs should adopt approaches that are differentiated from those used in general medical settings, emphasising personalised and context-specific strategies (Kim et al., 2024; World Health Organisation, 2021).

As described above, given the clinical complexity of residents in NHs, infection control efforts are best coordinated through interdisciplinary collaboration (Lee et al., 2024b; World Health Organisation, 2021). Within these teams, nurses play a pivotal frontline role, being primarily responsible for direct care, early detection of infection, and ongoing health monitoring. They often act as central coordinators in infection control processes, integrating information from multiple disciplines to maintain continuity and consistency in care delivery within NHs, where nurses frequently assume leadership functions in implementing and monitoring infection prevention programmes (Lee et al., 2024a). During emergencies, other team members commonly report through nurses, reflecting their central communicative and operational position within the care team.

Building upon the central role of nurses, infection control in NHs can be effectively grounded in an integrated approach that combines collaboration among diverse professionals with continuous education. To achieve this, NHs require a coordinated infection management system, as recommended in international infection prevention and control (IPC) frameworks (World Health Organisation, 2021). In this context, nurses serve as the anchor of interdisciplinary practice, sharing resident-specific infection control knowledge with the team and supporting collaborative and person-centred care strategies (Choi et al., 2025). Thus, a collaborative practice perspective—developed through interdisciplinary communication and ongoing education—can be embedded throughout infection control in NHs. Recent trends in infection control education have increasingly emphasised interdisciplinary curricula, underscoring the importance of team-based preparedness across long-term care settings (Chang et al., 2023; Choi et al., 2025; Hong et al., 2024).

Despite the growing importance of infection control, most research has continued to focus on acute care settings, with relatively limited attention to NHs and other long-term care environments (Navarro-Prados et al., 2024). Infection management in NHs is inherently multifaceted, requiring consideration not only of medical symptoms but also of residents’ psychological and social conditions (Choi et al., 2025). Given the prevalence of multiple comorbidities, functional limitations, and cognitive impairments in this population, infection control must be individualised and guided by interdisciplinary consensus that reflects each resident’s functional status and care needs (Cappelli et al., 2024; Cho et al., 2023; Tropea et al., 2023). In this context, recent Western studies have explored infection prevention and control in long-term care from an interdisciplinary perspective, shedding light on key issues such as team-based coordination, communication, and ethical challenges during the COVID-19 pandemic (Connelly et al., 2023; Crnich, 2022; Festa et al., 2023). However, while these studies have advanced structural and organisational understanding, few have examined how practitioners themselves conceptualise and make sense of infection control in practice. Understanding these practitioner perspectives is crucial for bridging the gap between institutional policies and everyday care delivery. Building upon this growing body of work, there remains a need to establish empirical evidence on how interdisciplinary practitioners in NHs conceptualise and enact infection control within their unique care contexts. While this study focuses on the South Korean long-term care context, its findings offer transferable insights for other countries experiencing similar demographic shifts and long-term care challenges (Organisation for Economic Co-operation & Development, 2023; World Health Organisation, 2021).

This study adopts a phenomenographic approach, which is well suited to exploring how interdisciplinary practitioners in NHs understand and enact infection control in practice. This approach enables the identification of qualitatively distinct ways of conceptualising infection control, providing insights into variations in practitioners’ meaning-making and into how interdisciplinary collaboration shapes infection management in real-world long-term care settings. Emerging infectious diseases and unpredictable pandemics (e.g., COVID-19) have underscored both the vulnerability of NH residents and the inherent complexity of infection control in these settings, thereby highlighting the urgent need for empirical research grounded in practitioners’ real-world experiences. Recent developments in gerontological education and practice further emphasise the importance of understanding practitioners’ perspectives and meaning-making processes as a foundation for improving infection control strategies and educational programmes (Connelly et al., 2023; Crnich, 2022; Festa et al., 2023). Therefore, this study aimed to explore how interdisciplinary practitioners in NHs conceptualise and enact infection control amid the COVID-19 pandemic, using a phenomenographic approach to reveal the qualitatively different ways in which infection control is understood and practiced.

### Study aim

This study aimed to explore how interdisciplinary practitioners—including nurses, physical therapists, and social workers—conceptualise infection control in nursing homes during the COVID-19 pandemic, and to identify the structural outcome space representing qualitatively different ways of understanding, with particular attention to the central role of nurses.

## Methods

### Study design

Phenomenography is a qualitative research approach that explores the qualitatively different ways people understand, experience, and conceptualise a given phenomenon (Barnard et al., 1999; Marton, 1981). Its focus lies not on the phenomenon itself, but on how individuals perceive and relate to it. These varying conceptions reflect the relationship between the subject and the phenomenon and are typically characterised by two dimensions: the “what” aspect, referring to the object of focus, and the “how” aspect, indicating how meaning is constructed (Larsson & Holmström, 2007; Yoon et al., 2023). Rather than classifying the content of responses, phenomenographic analysis seeks to map the variation in how people make sense of a phenomenon through empirical data. It is concerned with people's conceptions—not the ontological reality of the world—and aims to reveal the structural differences in how individuals define and interpret their experiences (Sjöström & Dahlgren, 2002). This methodological approach has been widely applied in health and education research, including studies involving interdisciplinary healthcare staff (Larsson & Holmström, 2007).

Accordingly, the present study employed a phenomenographic approach situated within a relational epistemology that is broadly aligned with a constructivist paradigm, which understands knowledge as relationally constituted through participants’ experiences and interactions with the phenomenon (Marton, 1981; Sjöström & Dahlgren, 2002), and was reported in accordance with the Standards for Reporting Qualitative Research (SRQR) (O’Brien et al., 2014).

### Participants and setting

In qualitative research, purposive sampling is considered an appropriate strategy for enhancing data quality and for exploring the diverse ways individuals experience a specific phenomenon (Creswell & Poth, 2018; Palinkas et al., 2015). For this reason, purposive sampling is commonly employed in phenomenographic research to identify participants who have directly experienced the phenomenon under investigation (Boon et al., 2007). When selecting participants, phenomenographic researchers must establish clear inclusion criteria to obtain rich and varied data (Hajar, 2021). Although the optimal number of participants has not been definitively determined, previous studies suggest that 15 to 20 participants are typically sufficient to capture a range of human experiences and variations in perception (Hajar, 2021; Trigwell, 2000). Following qualitative research design guidelines proposed by Creswell and Poth (2018), sampling continued until conceptual saturation was achieved—that is, when no new themes or variations in understanding emerged from additional interviews.

Accordingly, a purposive sample of 21 interdisciplinary practitioners (11 nurses, 5 social workers, and 5 physical therapists) from three nursing homes in South Korea was recruited as key informants capable of providing rich, practice-based insights. Participants were recruited through cooperation with nursing home administrators, who distributed study invitations to eligible staff members. The researcher explained the study purpose and procedures to all potential participants, and written informed consent was obtained prior to data collection.

The inclusion criteria were: (a) practitioners who had been working in the nursing home for at least one year; (b) those directly involved in daily infection prevention and control activities; and (c) willingness to participate and share their professional experiences. The exclusion criteria were: practitioners without direct care responsibilities (e.g., administrative or temporary staff) or those with less than one year of nursing home experience.

To capture variation in perspectives rather than statistical representativeness, we selected three facilities of different sizes with varying numbers of residents. These facilities had admission capacities of 65, 80, and 270 residents, respectively, and all maintained interdisciplinary teams that included nurses, social workers, and physical therapists. Despite differences in external factors such as facility size and operational policies, this study focused on exploring infection control practices from the shared perspective of experienced practitioners that could be applied across various nursing home contexts. Both authors, gerontological nursing scholars with extensive experience in long-term care settings, engaged in ongoing reflexive discussions throughout data collection and analysis. They reflected on their professional backgrounds and potential preconceptions, and critically reviewed analytic interpretations to enhance reflexivity and ensure the trustworthiness of the findings.

Participant demographic characteristics are summarised as follows. The average age of the 21 interdisciplinary participants was 43.2 years (range: 25–65 years). Two were male and 19 female, and their average working experience in nursing homes was 5.7 years (Table I).

**Table I.** General characteristics of interdisciplinary practitioners (n = 21).

No.	Occupation	Gender	Age (years)	Education	Total working experience (years)	Experience in NHs (years)
1	Nurse	Female	48	Bachelor’s degree	15 years	2 years
2	Nurse	Female	55	Bachelor’s degree	10 years	4 years
3	Nurse	Female	65	Bachelor’s degree	34 years	1 year
4	Nurse	Female	30	Bachelor’s degree	7 years	1 year
5	Nurse	Female	53	Master’s degree	21 years	4 years
6	Social worker	Female	34	Bachelor’s degree	10 years	8 years
7	Nurse	Female	51	Bachelor’s degree	15 years	8 years
8	Nurse	Female	29	Bachelor’s degree	5 years	4 years
9	Nurse	Female	50	Master’s degree	4 years	16 years
10	Nurse	Female	55	Bachelor’s degree	16 years	6 years
11	Nurse	Female	56	Master’s degree	6 years	4 years
12	Physical therapist	Female	38	Bachelor’s degree	7 years	7 years
13	Physical therapist	Female	28	Bachelor’s degree	2 years	2 years
14	Physical therapist	Male	41	Master’s degree	12 years	12 years
15	Physical therapist	Female	28	Bachelor’s degree	2 years	2 years
16	Social worker	Female	49	Bachelor’s degree	10 years	10 years
17	Social worker	Female	25	Bachelor’s degree	2 years	2 years
18	Social worker	Female	34	Bachelor’s degree	6 years	6 years
19	Social worker	Male	42	Bachelor’s degree	12 years	12 years
20	Nurse	Female	44	Master’s degree	5 years	5 years
21	Physical therapist	Female	53	Bachelor’s degree	4 years	4 years

NH: nursing home.

### Data collection

In qualitative phenomenographic research, key data are obtained through in-depth interviews with participants, continuing until conceptual saturation is reached. In this study, the concept of COVID-19 infection control in nursing homes was introduced to interdisciplinary practitioners, and clinical scenarios were used to explore their understanding of the phenomenon.

To stimulate practitioners’ conceptualisations and evoke reflections grounded in real-world situations, four clinical scenarios were developed based on actual care experiences. Rather than presenting generalised textbook knowledge, these scenarios were designed to help participants recall and interpret their own experiences. They served as realistic stimuli, consistent with phenomenographic principles that emphasise triggering perception through concrete situations (Åkerlind, 2005; Bowden, 2000; Marton, 1986).

Because it was impossible to reproduce actual COVID-19 infection control situations in NHs, realistic simulation scenarios were developed based on interviews with practitioners to simulate pandemic-related challenges. During the interviews, participants were shown these scenarios and were asked to recall and describe how they had managed infection control under similar circumstances (Marton, 1981; Yoon et al., 2023). Specifically, the scenarios were developed using findings from prior classification research on NH residents’ functional levels, preliminary interviews with NH practitioners, and a review of infection control literature and training materials (Im et al., 2014).

This approach ensured that the scenarios captured a range of physical and cognitive functioning among residents, allowing for the exploration of diverse experiences related to infection control during the COVID-19 pandemic. A summary of the scenario characteristics, including content, functional profiles, and clinical foci, is provided in Table II. The first scenario described a resident with mild dementia who was ambulatory and independent in daily activities. The second scenario depicted a physically healthy resident with moderate to severe dementia, exhibiting cognitive decline and behavioural disturbances. The third scenario involved a resident with mild dementia who could transfer from bed to wheelchair with partial assistance. The fourth scenario represented a bedridden resident with severe dementia who was unable to communicate verbally. Each scenario was designed to represent a distinct combination of physical and cognitive functioning relevant to infection control decision-making in NHs. The content validity of the scenarios was reviewed by seven experts, including five NH directors with over 29 years of clinical experience and two professors of gerontological nursing with over 15 years of clinical experience and at least six years of academic experience. The experts confirmed the realism, relevance, and applicability of the scenarios as effective tools for eliciting practice-based insights.

**Table II.** Clinical scenarios for COVID-19 infection control with distinct physical and cognitive foci among NH residents.

Scenario No.	Contents of the scenarios	Distinct functions	Focus
1	Elderly A (F/80) walks independently and well on her own, but her dementia prevents her from remembering anything over time. She also has some orientation to time, place, and person and is able to carry out daily life activities. She came out to the facility’s living room wearing a mask, thinking about the importance of wearing a mask, which she had learned about in the NH's programme yesterday. However, perhaps because she felt instinctively uncomfortable, she quickly took off her mask, threw it on the floor, and walked around humming songs.	Mild dementia, Ambulatory	Refusal to wear a mask despite preserved cognitive function
2	Although elderly B (M/82) is physically healthy, he shows serious problematic behaviour such as wandering. He speaks in a personal language and produces meaningless repetitive sounds, making everyday conversation impossible. Interacting with other people is nearly impossible for him. The resident does not comply, even though the nurse keeps telling him to wear a mask. Recently, a confirmed case of COVID-19 has occurred in NHs, so the risk of infection continues to be very high. To prevent the resident from becoming infected, a string was tied to the back of the mask. However, he quickly rips the mask strings and throws the mask away.	Severe cognitive decline, Physically active	Wandering behaviour and refusal to follow infection control instructions
3	Elderly C (M/85), who is hospitalised in a four-person room at a NH, has mild dementia and is able to move out of bed in a wheelchair and perform some daily activities with partial assistance from NH practitioners. However, after COVID-19, he mainly stays in bed, and has severe joint contractures, making it difficult for him to move. To prevent infection, the resident has been eating alone in his room with the curtain closed. A physical therapist comes to his room periodically and provides him ROM exercises on a one-to-one basis. Although he understands the dangers of infection, he absolutely refuses to wear a mask due to stubbornness arising from symptoms of dementia. While this resident has no fever, he has been coughing continuously for the past two days. The nurse immediately performed both a RATs and a PCR test, but so far, the results have been negative.	Mild dementia, Limited mobility	Immobility-related infection risk and refusal of preventive measures
4	Elderly D (F/91) suffers from severe dementia and has difficulty moving independently, even in bed. She is unable to react or express anything in particular, and just blinks while lying on the bed. The nursing care worker in room 1 had a sore throat and was not feeling well since yesterday, possibly due to the change of seasons. She went to the hospital, took prescribed cold medicine, and worked the night shift last night. The morning after her shift, she found her throat still sore and her voice hoarse. She immediately took a PCR test and received a positive result. The caregiver cared for Elderly D in room 1, at her bedside all night. Fortunately, the resident appears to have had no special COVID-19 symptoms so far.	Severe dementia, Bedridden	Non-verbal, fully dependent resident with high infection vulnerability

M: male, F: female, PCR: polymerase chain reaction, RATs: rapid antigen tests, ROM: range of motion.

A semi-structured questioning guide was developed according to phenomenographic principles (Marton, 1981) to elicit practitioners’ perceptions and reflections based on the four clinical scenarios. The guide consisted of four open-ended questions: (1) “How did you respond to infection control for a resident with this level of physical and cognitive functioning during the COVID-19 pandemic?”; (2) “What difficulties did you experience in implementing infection prevention measures in this situation?”; (3) “What types of collaboration were necessary with other staff members when managing infection control?”; and (4) “What factors influenced your infection control decision-making in such situations?” Follow-up probing questions were used as needed to further clarify or deepen participants’ explanations (Yoon et al., 2023).

Data were collected through one-on-one in-depth interviews with 21 interdisciplinary practitioners across three NHs in February and March 2023. Depending on availability, each participant was interviewed once or twice. In accordance with NH protocols, the researcher implemented preventive infection control measures and preemptive COVID-19 testing before conducting face-to-face interviews with 17 participants. Due to infection control concerns, the remaining four participants were each interviewed once by telephone.

All interviews were conducted and transcribed in Korean, as all participants were native Korean speakers. The original transcripts were analysed to preserve contextual meanings. Selected quotations were later translated into English by a bilingual qualitative researcher and reviewed by another bilingual expert to ensure both linguistic and conceptual accuracy.

### Data analysis

Data were analysed following the seven-step phenomenographic approach by Dahlgren and Fallsberg, (1991). The researcher repeatedly read the transcripts to gain familiarity with the data, identified and condensed meaningful statements, and grouped similar responses into preliminary categories. These were then refined and named to capture their essential meanings. Finally, categories were compared to identify both their unique characteristics and interrelationships. An audit trail documenting analytic decisions and category development was maintained throughout the process to ensure transparency and allow verification of the analytical procedure.

To ensure rigour and analytical consistency, the study applied multiple verification strategies grounded in phenomenographic rigour (Åkerlind, 2005; Marton, 1986; Sandberg, 1997). Data coding was independently conducted by two researchers—the first author and a qualitative nursing research expert experienced in phenomenographic methodology. Both researchers reviewed the transcripts and coding framework separately, and any discrepancies or interpretive differences were discussed through iterative comparison until consensus was reached. When complete agreement could not be achieved, a third expert in qualitative nursing research was consulted, and final decisions were made through a consensus meeting. Peer debriefing sessions were also conducted to critically review category interpretations and enhance reflexivity. This multi-step verification process enhanced analytical rigour and strengthened the overall credibility of the findings. Furthermore, these approaches ensured communicative validity (through collaborative dialogue), pragmatic validity (through applicability to practice), and reliability (through transparent documentation and peer review), in line with phenomenographic methodological principles.

The resulting categories were structured into an outcome space, which visually represents the logical and hierarchical relationships among practitioners’ qualitatively different ways of understanding the phenomenon (Barnard et al., 1999).

### Rigour and trustworthiness

Rigour and trustworthiness were ensured through strategies grounded in phenomenographic rigour (Åkerlind, 2005; Marton, 1986; Sandberg, 1997). Communicative validity was achieved through continuous dialogue among researchers and peer debriefing to test the coherence and logical consistency of the categories and the outcome space. Pragmatic validity was established by examining the applicability and relevance of the findings to practice and educational contexts. Reliability was maintained through transparent documentation of analytic decisions, iterative comparison, and the use of an audit trail.

To ensure semantic and conceptual accuracy in translation, bilingual experts reviewed the English quotations against the original Korean transcripts. The research team cross-checked all translations and resolved any ambiguous expressions through discussion until full consensus was reached.

## Results

**Figure 1.**
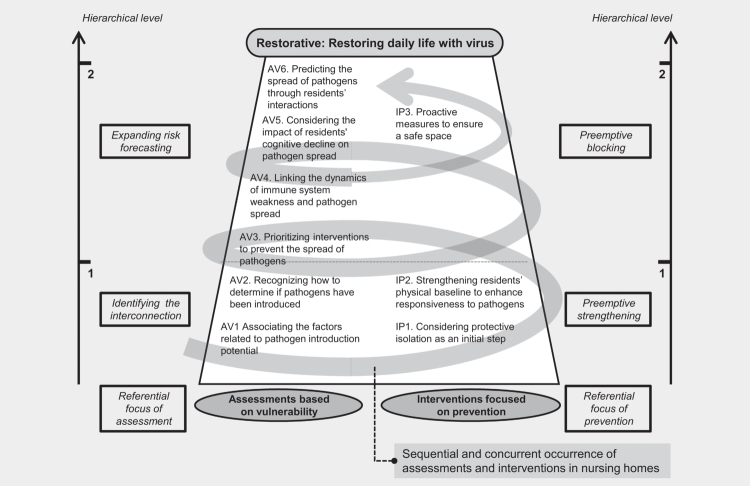
Outcome space derived from an interdisciplinary perspective, illustrating how nursing home practitioners conceptualise infection control during COVID-19. The outcome space is structured around two referential foci—assessment based on vulnerability (AV) and intervention focused on prevention (IP)—and visualises the hierarchical relationships among descriptive categories.

Figure 1 presents the overall conceptual structure and key findings of this study, visualising the hierarchical relationships between categories in the derived outcome space. It reflects how interdisciplinary practitioners, who directly experienced COVID-19 management in NHs, conceptualised infection control through practice grounded in real-world experience, alongside theoretical understanding. Two interrelated category groups emerged: assessments based on vulnerability (AV) and interventions focused on prevention (IP). These categories reveal how practitioners perceived infection control as a dynamic and ongoing process, involving continuous risk assessment and proactive response. Figure 1 visualises these hierarchical relationships, while Table III presents the detailed sub-categories and illustrative quotations that ground these conceptualisations in actual clinical practice.

**Table III.** Summary of descriptive categories and illustrative quotations.

Groups	Categories	Relevant quotations from the interviews
Assessments based on vulnerability	AV1. Associating the factors related to pathogen introduction potential	There were two bedridden residents in one room. The facility staff thought that since the residents stayed in bed, there was no chance of infection. However, the care helper caring for that room had a cold symptom and was found to be positive for COVID-19 by a RATs. In addition, a PCR test was conducted to confirm the diagnosis, and the results were being waited for. During this period, one of the residents in the room cared for by the care helper showed symptoms of fever and hoarseness. In the end, he was confirmed to have the COVID-19 virus. [*NS with 8 years of NH experience*]
	AV2. Recognising how to determine if pathogens have been introduced	When a nurse puts a mask on a resident with moderate dementia, he takes the mask off within a few seconds and leaves his room and walks around. He had tested positive for the COVID-19 and was supposed to stay in his room, but he kept forgetting about the infection situation and came out, coughing and trying to move around. He had symptoms of wheezing, and a RATs was performed as soon as an infection was suspected. Two patients who were in close contact with him in the same room also underwent tests immediately. Although their results were negative, they were quarantined in a different room for a few days and observed for infection symptoms. [*NS with 4 years of NH experience*] A resident who had never had a fever started exhibiting a fever one day. A RATs was conducted and the results were positive, so she was immediately placed alone in a temporary isolation room. Perhaps because she was a resident with mild dementia who was able to walk freely and carry out daily activities independently, she refused to be left alone in the isolation room no matter how much the staff explained the current situation. So, a nurse who had recently been infected with COVID-19 was deliberately placed with the resident in the room and provided her with 1:1 care. Afterwards, the patient became stable and did not try to leave the room at all, and there was no further spread of infection within the facility. [*NS with 4 years of NH experience*]
	AV3. Prioritising interventions to prevent the spread of pathogens	There had been no infections at the facility for some time. However, because there may be asymptomatic infected people living in the facility, practitioners and elderly residents have been conducting PCR tests periodically. Even now, periodic inspection schedules for all residents are planned during an interdisciplinary management meeting within the facility. If it has been a while since the PCR test was performed, managers set a test date and perform a full PCR test. [*SW with 8 years of NH experience*]
	AV4. Linking the dynamics of immune system weakness and pathogen spread	There was a bedridden resident with severe dementia who needed full assistance and was unable to communicate hospitalised in a four-person room. None of the staff or residents on the same floor were confirmed positive, but the elderly person was infected with COVID-19. As a result of the epidemiological investigation, it was revealed that there were several face-to-face meetings and several deliveries of goods at that time. Facility staff were surprised to learn that even if everyone stayed in their designated space, a bedridden resident with low energy could be vulnerable to infection. After that, the staff wore stronger gloves when coming into contact with or providing care to the elderly and paid more attention to disinfecting their surroundings. [*PT with 7 years of NH experience*] Last Christmas, the government allowed in-person visits at facilities. After face-to face visits began, residents on several floors suddenly tested positive for COVID-19 and were quarantined in cohorts. Before the visitations, the staff was infected first and then the elderly, but this time, the staff was not infected and only the elderly were infected. [*NS with 6 years of NH experience*] Elderly people with reduced functions are not in a position to defend themselves against infection. They can become infected through caregivers, such as facility staff or guardians, regardless of their will. In particular, the resident in a bedridden state has poor basic health status. If they become infected, their health can seriously deteriorate and even lead to sudden death. Thus, facility staff must thoroughly carry out infection control activities. [*NS with 6 years of NH experience*]
	AV5. Considering the impact of residents' cognitive decline on pathogen spread	A resident with moderate to severe dementia who wandered would go into another room anytime the staff took their eyes off her. Because elderly people in a bedridden state cannot move at all, they pose little risk of spreading infection, but elderly people who walk well become spreaders of infection. Because human-to-human contact is the most dangerous transmission vehicle for COVID-19, the staff have no choice but to continuously monitor it, and supervision of the movement of the elderly is most necessary. [*NS with 1 year of NH experience*] There was a resident wandering around the facility’s living room in a wheelchair. When it was summer and he felt out of breath, he took off his mask and threw it away. Sometimes, he kept the mask in his pocket, a symptom similar to the hoarding disorder of an elderly person with dementia. In the past, because older people used things sparingly, they seemed to fold clean masks carefully and store them in their clothes pockets. When we did laundry, we found several masks in his clothes pockets. [*SW with 6 years of NH experience*]
	AV6. Predicting the spread of pathogens through residents’ interactions	I think the most important thing was to minimise the opportunities for contact or interactions that could spread the coronavirus. Just as cohort isolation was effective in blocking the spread of infection, in severe cases of COVID-19, physical therapy was performed on only one floor per day to avoid overlapping movement lines as much as possible. For those in a bedridden condition, a physical therapist visited the room to perform physical therapy. For those in a four-person room, we kept the distance between beds as great as possible and went inside the curtains to provide physical therapy to reduce interactions. [*PT with 12 years of NH experience*]
Interventions focused on prevention	IP1. Considering protective isolation as an initial step	There was a resident with dementia and diabetes who was hospitalised in a single room. He did not eat as much as he wanted to, perhaps because he was afraid that his blood sugar level would rise after eating. Also, after eating, he kept spitting the food out and vomiting on purpose, perhaps out of fear of a high blood sugar level. Fortunately, he stayed alone in a single room, so staff were less worried about the spread of infection. The care worker in charge wore disposable gloves, immediately threw the remaining food he spat out into the toilet, and disinfected her hands. She sprayed disinfectant around the resident and always strengthened disinfection and ventilation in the bathrooms, including the toilets. [*NS with 4 years of NH experience*]
	IP2. Strengthening residents’ physical baseline to enhance responsiveness to pathogens	Even residents with mild dementia cannot control wearing masks. They understand why they need to wear a mask, and they answer with a smile, but they don't put anything into practice. So, when several people were infected with COVID-19, we separated the floors, like in a cohort quarantine, and had the residents only move around within the unit. Fortunately, there wasn't a single elderly person in the unit in a serious condition, so everyone infected suffered from it like a really light cold. Since all the residents were in good health, the practitioners had faith that they would get through it without any major deterioration. [*PT with 4 years of NH experience*]
	IP3. Proactive measures to ensure a safe space	Our facility has changed its operation to quasi-cohort quarantine when a person is infected. Because movement between wards, floors, and rooms was completely prohibited, COVID-19 did not spread to a large extent. [*SW with 8 years of NH experience*]

BPSD: behavioural and psychological symptoms of dementia, NH: nursing home, NS: nurse, PT: physical therapist, SW: social worker, PCR: polymerase chain reaction, RATs: rapid antigen tests.

### Assessments based on vulnerability (AV)

Practitioners emphasised that effective infection control in NHs during pandemic outbreaks requires careful assessment of residents’ physical and cognitive functions. As frontline caregivers, they are often the first to detect subtle clinical changes that diagnostic tools may overlook. This category group underscores that meaningful assessment extends beyond symptom detection to include physical frailty, cognitive decline, and the interaction of environmental and structural factors within the facility.

#### AV1. Associating the factors related to pathogen introduction potential

This category captures how practitioners associated residents’ physical mobility with the potential routes of pathogen introduction within NHs. The route of infection was perceived to vary depending on residents’ physical mobility. Residents who were bedridden and room-bound were typically infected through contact with staff members entering their rooms, whereas ambulatory residents often contracted infections independently through movement within shared spaces. As one nurse noted, “Even residents who stayed in bed became infected after a caregiver with a mild cold tested positive” (Nurse, 8 years of NH experience). These experiences illustrated that mobility itself was recognised as a potential risk factor for pathogen introduction within the facility.

#### AV2. Recognising how to determine if pathogens have been introduced

Diagnostic testing was considered essential for confirming suspected cases. Practitioners highlighted the complementary use of rapid antigen tests (RATs) for immediate screening and polymerase chain reaction (PCR) testing for confirmation. As one practitioner stated, “When a resident kept coughing and wandering, we tested immediately and confirmed infection with RATs and PCR” (Nurse, 4 years of NH experience). Elderly residents often exhibited atypical symptoms—such as changes in voice, increased sputum, or respiratory difficulty—before fever onset, which required vigilant monitoring.

#### AV3. Prioritising interventions to prevent the spread of pathogens

Infection control strategies were flexibly adjusted according to whether COVID-19 had been confirmed within the facility. Before any infections were detected, regular PCR testing was planned as a preventive measure to identify possible asymptomatic carriers in advance, while after an outbreak, practitioners focused on physical separation and cohorting of residents to block further transmission. As one social worker described, “We regularly planned PCR testing schedules during interdisciplinary meetings to prevent infection in advance” (Social worker, 8 years of NH experience).

#### AV4. Linking the dynamics of immune system weakness and pathogen spread

Residents with weakened immune systems—due to chronic comorbidities or invasive devices such as feeding tubes, Foley catheters, or percutaneous endoscopic gastrostomy (PEG)—were identified as high-risk individuals. Practitioners stressed the need for advanced medical and functional assessments in these populations, noting that even after recovery, long-term complications such as reduced appetite, fatigue, contractures, pressure ulcers, and cognitive decline often remained. As one physical therapist stated, “Elderly residents with underlying conditions are vulnerable to infections, and if they become infected, their health can deteriorate rapidly and even lead to sudden death” (Physical therapist with 7 years of NH experience).

#### AV5. Considering the impact of residents’ cognitive decline on pathogen spread

Practitioners noted that behavioural and psychological symptoms of dementia (BPSD) are manageable under normal conditions but significantly increase infection risks during infection outbreaks. As one nurse stated, “Ambulatory residents with dementia are a major source of infection spread in infection situations” (Nurse, 1 year of NH experience). Wandering and repetitive abnormal behaviours increased cross-contact and exposure to infectious secretions. Residents exhibiting such behaviours were managed through close, individualised supervision.

#### AV6. Predicting the spread of pathogens through residents’ interaction

Minimising opportunities for contact or interaction among residents was recognised as a key infection control strategy during COVID-19 outbreaks. Practitioners adjusted schedules for physical therapy and daily activities to prevent overlapping movement lines and cross-unit contact. As one physical therapist noted, “We performed physical therapy on only one floor per day and visited bedridden residents in their rooms to minimise interactions” (Physical therapist, 12 years of NH experience). These proactive strategies reflected practitioners’ efforts to anticipate and block potential routes of pathogen transmission within nursing homes.

### Interventions focused on prevention (IP)

Based on the practical knowledge gained and refined directly by interdisciplinary practitioners during the COVID-19 pandemic, three core prevention-focused strategies were identified. These strategies were not abstract theories but real-time solutions developed and adapted on-site under rapidly changing conditions. The interventions reflect how practitioners, faced with uncertainty and risk, implemented concrete infection control measures rooted in their daily caregiving experiences.

#### IP1. Considering protective isolation as an initial step

Practitioners emphasised that infection control must start with immediate and consistent protective isolation—before, during, and even after a confirmed infection. From a preventive standpoint, they also reinforced disinfection and ventilation preemptively, treating these actions as part of protective isolation. In high-risk moments, strict hygiene protocols were instinctively followed: not only handwashing and masking, but also donning full protective gear such as gowns and face shields. As one nurse shared, “We strengthened disinfection and ventilation in the rooms and bathrooms, especially for residents showing infection-related behaviours such as spitting or vomiting” (Nurse, 4 years of NH experience). Bedridden residents, who couldn’t protect themselves, were prioritised for careful and ethical infection control. Many practitioners noted they would voluntarily test themselves using rapid antigen tests and self-isolate at home for 1–2 days at the slightest sign of symptoms—an informal but vital measure to safeguard residents.

#### IP2. Strengthening residents’ physical baseline to enhance responsiveness to pathogens

Practitioners agreed that elderly residents who were healthier to begin with—well-nourished, mobile, and functionally stable—were better able to recover from COVID-19. As one physical therapist described, “Even residents with mild dementia couldn’t always manage masks properly, but because everyone was in relatively good condition, those infected recovered like from a light cold” (Physical therapist, 4 years of NH experience). Even when infected, some residents responded to symptomatic care much like recovering from a mild illness. In contrast, those already weakened—especially with pressure ulcers or multiple catheters—often declined rapidly, with some dying unexpectedly. To prevent such deterioration, practitioners took active steps to reinforce residents’ baseline strength by ensuring adequate nutrition, preventing weight loss, and protecting skin integrity—viewing these efforts not as routine care but as infection prevention strategies in themselves.

#### IP3. Proactive measures to ensure a safe space

This category reflects how practitioners proactively redesigned NH spaces to minimise transmission risk during outbreaks. From the very start of an outbreak, practitioners responded swiftly by restructuring the physical space of the facility. Nursing homes were reorganised into “clean,” “semi-contaminated,” and “contaminated” zones. Movement routes were reviewed and adjusted; doors were closed off, temporary walls were installed, and care schedules were redesigned by ward or floor. As one social worker explained, “Our facility changed its operation to quasi-cohort quarantine when a person was infected. Because movement between wards, floors, and rooms was completely prohibited, COVID-19 did not spread to a large extent” (Social worker, 8 years of NH experience). These adaptive spatial strategies—born directly from frontline problem-solving—were repeatedly cited as key to containing the virus within specific areas and preventing widespread transmission.

### Disciplinary emphasis within interdisciplinary practice

While all practitioners shared a collective commitment to infection control, distinct emphases emerged across professional groups, reflecting their unique roles and perspectives. Nurses most frequently focused on early detection and risk containment, emphasising real-time clinical judgement in response to subtle changes in residents’ conditions. Their accounts often highlighted the instinctive use of protective isolation and proactive testing even before formal diagnosis—underscoring their frontline role as infection sentinels. In contrast, physical therapists repeatedly emphasised the importance of preserving residents’ baseline function. They viewed physical decline not only as a health concern but also as a risk factor for infection vulnerability, and described how individualised rehabilitation scheduling minimised transmission opportunities while maintaining mobility. Social workers, meanwhile, drew attention to the behavioural risks posed by cognitive impairment—such as wandering and mask removal—and stressed the need for psychosocial strategies and vigilant supervision to mitigate BPSD-related exposure.

These recurring emphases illustrate how interdisciplinary practitioners co-constructed infection control as both a shared and differentiated practice. By capturing these profession-specific insights, the study not only deepens understanding of how infection control unfolded in practice but also provides a grounded foundation for role-sensitive, team-based educational strategies.

### Structure of the outcome space

Figure 1 illustrates the outcome space, showing how interdisciplinary NH practitioners responded to the abrupt emergence of infection during the COVID-19 pandemic. This framework reflects not theoretical abstractions but grounded, field-driven insights accumulated by practitioners through firsthand crisis experience. The structure emerged from their evolving sense-making—what they assessed, how they responded, and in what order they acted.

The outcome space is organised around two referential domains: assessments based on vulnerability (AV) and interventions focused on prevention (IP). These domains are not discrete but interlinked, mirroring the fluid and cyclical nature of real-world infection control. Each domain is structured by a clear hierarchy—from early-stage detection to containment—while also reflecting a deeper conceptual duality: the *“what”* (the content of infection control) and the *“how”* (the strategies of action).

In the assessment based on vulnerability (AV), the referential foci were “identifying the interconnection” and “expanding risk forecasting”. Practitioners began by identifying functional and environmental factors that heightened the likelihood of viral entry—such as residents’ mobility, external contact points, and staff interactions. Once suspected, they moved swiftly to observe early symptoms and conduct rapid diagnostic testing (e.g., RATs and PCR). As the virus spread, their attention shifted to identifying asymptomatic cases and evaluating immune vulnerability among residents—especially those with comorbidities or dependent on catheters. Practitioners also evaluated how BPSD in cognitively impaired residents contributed to viral transmission through wandering or abnormal oral behaviours. Finally, they considered the degree of resident interaction as a determinant of how far and fast the virus could spread.

In the intervention focused on prevention (IP), the referential foci were “preemptive strengthening” and “preemptive blocking”. Preemptive strengthening involved maintaining a constant state of readiness—rigorous hand hygiene, personal protective equipment (PPE) use, routine disinfection, and promoting resident health through nutrition and physical resilience. This was not done only after infection but as a sustained baseline to improve outcomes if infection occurred. When cases emerged, practitioners swiftly enacted movement restrictions and spatial separations, converting wards into zones and erecting physical barriers. These actions were based not on manuals but on dynamic problem-solving in the moment—measures refined with each new wave.

Together, these assessment and intervention-based processes form a structured yet adaptable model. The outcome space is not static but dynamic—reflecting an evolving, practice-based logic shaped by lived experience during the pandemic. Ultimately, it provided a framework through which NH residents—who must live with the possibility of infection—could avoid further functional decline and, in many cases, recover their routines and dignity.

## Discussion

The COVID-19 pandemic exposed the vulnerability of infection control systems in NHs, where older adults became some of the most severely affected (Giri et al., 2021). Unlike acute care settings with constant medical oversight, NHs often lack on-site physicians and instead rely on interdisciplinary teams led by nurses to manage infectious disease outbreaks (D’Souza et al., 2024). While interdisciplinary studies have explored infection control in long-term care, their findings have often remained at a conceptual level, with limited practical applicability to the realities of NH practice (Connelly et al., 2023; Crnich, 2022; Festa et al., 2023). This study is one of the first to phenomenographically explore and conceptualise NH practitioners’ perceptions of infection control, based on firsthand experiences during the pandemic. Crucially, the pandemic is not a concluded event but an ongoing global concern (World Health Organisation, 2024), and the practical knowledge developed and refined on the frontlines of NHs represents a vital asset for future preparedness. The experiential insights captured in this study offer not only an empirical foundation for better understanding NH infection control practices but also a meaningful contribution to future care strategies that protect vulnerable populations in similar high-risk environments.

Our findings indicate two interrelated domains that capture how interdisciplinary practitioners understood infection control in NHs during the COVID-19 pandemic: AV and IP. The AV domain focused on identifying residents’ physical, cognitive, and environmental vulnerabilities, whereas the IP domain described proactive measures designed to prevent and contain infections. Together, these findings suggest that infection control operates as a dynamic and cyclical process of continual risk recognition and preemptive intervention—underscoring nurses’ central coordinating role and the importance of collaborative, practice-based approaches to safeguarding frail older adults.

Although several qualitative studies have attempted to develop infection control strategies during the COVID-19 pandemic (e.g., Huang et al., 2021; Konetzka et al., 2021), most remained within pre-existing theoretical frameworks and faced limitations in practical application to long-term care settings. This study addresses those gaps through three key strengths: (1) the selection of NHs that demonstrated resilience through effective K-quarantine practices; (2) participation by interdisciplinary practitioners with direct, frontline experience during the pandemic (with over one year of experience); and (3) a targeted focus on frail elderly residents with physical and cognitive decline. K-quarantine practices refer to Korea’s nationally coordinated infection control strategy, which emphasised proactive testing, contact tracing, isolation of cases and contacts, and information and communications technology (ICT)-based surveillance systems (Choi et al., 2022; Ministry of Foreign Affairs of the Republic of Korea, 2020). Rather than implementing nationwide lockdowns, this strategy focused on the rapid identification and containment of COVID-19 cases at both community and institutional levels. Unlike previous research, which has largely focused on nurses in acute care settings, this study highlights a practice-based, interdisciplinary approach grounded in the everyday realities of chronic care facilities. It contributes novel insight into how infection control strategies can be adapted to support the daily lives and health maintenance of highly vulnerable older adults.

A major contribution of this study lies in its integrated analysis of both the commonalities and distinctions among interdisciplinary practitioners involved in infection control. Practitioners worked collaboratively toward the shared goals of preventing infection spread and preserving residents’ functional abilities, while fulfilling complementary roles grounded in their respective expertise. This interdisciplinary, team-based approach aligns with international recommendations for coordinated infection prevention and control systems in long-term care settings (World Health Organisation, 2021). Nurses led the early recognition of infection signs and initiated timely interventions (Abdalhafith et al., 2025); physical therapists continued rehabilitative efforts to maintain residents’ physical function even under isolation (Negm et al., 2022); and social workers supported residents’ psychological well-being and facilitated communication with families to mitigate the effects of social disconnection (Richardson et al., 2022). This role differentiation and coordination among professional groups functioned as a practice-based, integrative strategy that extended beyond traditional, manual-based infection control models (Chang et al., 2023). Notably, some categories reflected distinctive emphases depending on professional discipline. Nurses predominantly contributed to categories related to infection identification and medical risk assessment (e.g., AV2, AV4), whereas physical and occupational therapists emphasised strategies to sustain residents’ physical function and spatial reorganisation during outbreaks (e.g., IP2, IP3). Social workers, by contrast, were more involved in categories related to cognitive-behavioural risks and social interaction dynamics (e.g., AV6). These patterns further underscore the complementary nature of interdisciplinary collaboration in NH infection control.

Furthermore, this study offers a unique perspective on infection control in NHs during the COVID-19 pandemic by shifting the focus from the emotional burden of nurses—commonly emphasised in previous research—to the experiential, practice-based knowledge of interdisciplinary practitioners. By systematically organising how practitioners assessed risks and implemented interventions on the ground, this study presents a field-driven framework (Kim et al., 2023) for infection management that supports residents’ dignity and functional preservation during crises. These insights contribute not only to enhancing the effectiveness of infection control education and standardising training materials but also to building a practical knowledge base for future infectious disease preparedness. Moreover, by highlighting the collaborative yet differentiated roles of nurses, physical therapists, and social workers (Yoon et al., 2023), the study advances an integrated understanding of interdisciplinary infection control in long-term care.

Each descriptive category systematically conceptualises the complex and atypical characteristics of infection control in NHs and offers valuable insights into how interdisciplinary practitioners perceive and approach these challenges. The assessment and intervention processes revealed that care in NHs inevitably leans toward prevention-focused approaches—specifically, preactive and proactive nursing (Kim et al., 2023). Unlike acute care hospitals, NHs do not have physicians on-site at all times, and when an outbreak occurs, frail older residents may face life-threatening outcomes (Dyer et al., 2022). Therefore, preventive care is increasingly recognised as both a practical necessity and a guiding philosophy of care in NHs (Lee et al., 2024b; World Health Organisation, 2021). In this interdisciplinary setting, nurses often take the lead in infection management, guiding and coordinating care. This central role aligns closely with the core values of nursing—protecting life, promoting health, and preventing harm—and reflects the essence of nursing as a profession grounded in proactive, person-centred practice (Cappelli et al., 2024).

At the assessment stage, practitioners moved beyond surface-level infection control knowledge to deeply investigate the core issues they had encountered in practice, grounded in the unique characteristics of the facility and the conditions of the residents (Halperin et al., 2025). Amid an unpredictable pandemic, they systematically identified older adults’ symptoms and complex infection patterns, and based on these insights, provided timely and appropriate interventions (Nilsen et al., 2024). Unlike previous research, which often focused on standardised protocols, this study highlights the practical knowledge developed on the ground. Practitioners dynamically tailored therapeutic and supportive care environments and flexibly adjusted their responses to the shifting realities of the pandemic (D’Souza et al., 2024; Kim et al., 2024).

This study identified two main descriptive categories and an outcome space that reflect the dynamic, cyclical nature of infection control in NHs. In these settings, effective care requires continuous interplay between assessment and intervention, rather than a linear approach. Interdisciplinary teams must assess evolving conditions and respond simultaneously to ensure that planned interventions are fully and appropriately implemented. Grounded in practitioners’ real-world experiences during the pandemic, these findings underscore the importance of flexible, coordinated care strategies aimed at protecting the dignity and autonomy of elderly residents (Organisation for Economic Co-operation & Development, 2023; World Health Organisation, 2021).

Above all, this study offers meaningful contributions from an educational perspective, particularly in strengthening the competencies of interdisciplinary teams including NH nurses. Given that phenomenography is rooted in educational theory, the referential foci derived from this study provide clear guidance on where to concentrate education in infection control. As pandemics like COVID-19 remain ongoing and new practitioners continue to enter NHs, standardised infection control education is increasingly recognised as essential for strengthening workforce preparedness (Organisation for Economic Co-operation & Development, 2023; World Health Organisation, 2021). This emphasis is consistent with global recommendations that call for enhancing IPC competencies in long-term care and health systems worldwide (Organisation for Economic Co-operation & Development, 2023; World Health Organisation, 2021). According to the WHO’s Infection Prevention and Control in Long-Term Care Facilities guidance, continuous IPC training and system-level preparedness are key to protecting vulnerable older populations. Likewise, the OECD emphasises the need for sustained investment in workforce capacity and resilience across long-term care and health systems to prepare for future public health crises. These findings thus serve not only as a valuable resource grounded in real-world practice, but also as a foundational stepping stone for developing interdisciplinary training programmes and evaluation tools. Because the framework derived from this study was developed through direct experiences of frontline practitioners, it offers transferable insights applicable to other aging societies that face similar institutional and demographic challenges. Furthermore, the implications of this study add to the global discourse on infection prevention and control in long-term care, aligning with international efforts by the WHO and OECD to enhance resilience and preparedness in aging societies. Moreover, this contribution extends beyond Korea and may offer insights for advancing gerontological nursing education globally in an aging society.

Although this study proposes generalised care strategies that may be applicable globally to advance infection control in long-term care settings, further research is recommended to account for diverse environmental and cultural factors—such as the ideology of filial piety and national consciousness—that may shape care practices in specific regions. This study provides a foundation for developing interdisciplinary infection control education programmes in NHs and for strengthening practitioners’ infection control competencies. It also offers a basis for enhancing the educational capacity of newly employed nurses. Moreover, longitudinal and cross-cultural comparative studies could deepen understanding of how practitioners’ conceptualisations of infection control evolve across diverse sociocultural and healthcare contexts. As the global elderly population continues to rise and new infectious diseases emerge, the findings of this study provide valuable guidance for educating interdisciplinary practitioners and developing practical care strategies. In particular, the inclusion of diverse discipline nurses, physical and occupational therapists, and social workers—enabled a more comprehensive and practice-oriented understanding of infection management, beyond traditional, manual-based models. Future comparative studies across Eastern and Western contexts may uncover additional insights and suggest new directions for improving infection control in NHs worldwide.

## Limitation

This study has certain limitations that should be acknowledged. The findings were derived from a relatively small number of nursing homes located in Korea, which may limit their transferability to other cultural or healthcare settings. Although two researchers independently analysed the data and reached consensus through discussion and expert consultation, potential bias may remain due to the researchers’ professional backgrounds. Moreover, while reflexive journaling and iterative comparison were employed to enhance analytical rigour, complete objectivity cannot be assured in qualitative interpretation. Despite these constraints, the study provides an in-depth and contextually grounded understanding of how interdisciplinary practitioners conceptualise infection control in nursing homes during pandemics. These limitations should be interpreted acknowledging that credibility, transferability, dependability, and confirmability can be enhanced—but never fully guaranteed—in qualitative enquiry.

## Conclusion

This study demonstrates that the safety and quality of life among NH residents are shaped by how interdisciplinary practitioners conceptualise and respond to idiopathic infections, organised through referential focus. The infection prevention and response strategies derived from practitioners’ real-world experiences offer valuable insights for interdisciplinary education, particularly for NH nurses. These findings support the development of tailored care plans that address the specific needs of frail older adults while promoting coordinated, person-centred care. The proposed framework provides a solid foundation for future training and education—especially for practitioners without prior pandemic experience—and offers practical guidance for strengthening infection control in long-term care settings.

## Data Availability

The data supporting the findings of this study are available from the corresponding author upon reasonable request. The data are not publicly available due to privacy and ethical restrictions.

## Manuscript category

Original research (Qualitative empirical study).
